# Aggregative chimeric multicellularity in the absence of lethal kin discrimination

**DOI:** 10.1093/ismejo/wrag136

**Published:** 2026-06-11

**Authors:** Michael L Weltzer, Pravas C Roy, Jack Govaerts, Daniel Wall

**Affiliations:** Department of Molecular Biology, University of Wyoming, 1000 E University Avenue, Laramie, WY 82071, United States; Department of Molecular Biology, University of Wyoming, 1000 E University Avenue, Laramie, WY 82071, United States; Department of Molecular Biology, University of Wyoming, 1000 E University Avenue, Laramie, WY 82071, United States; Department of Molecular Biology, University of Wyoming, 1000 E University Avenue, Laramie, WY 82071, United States

**Keywords:** myxobacteria, kin discrimination, type VI secretion system, fruiting body development

## Abstract

Aggregative multicellularity is a cooperative strategy used by some microbes. Unlike plant and animal development, which proceeds through clonal expansion from a single progenitor cell, aggregation is susceptible to genetic conflict and cheating, threatening multicellular stability. *Myxococcus xanthus* and *Dictyostelium* spp. are soil-dwelling models that form spore-producing fruiting bodies by aggregation upon starvation. Studies of natural *M. xanthus* fruiting bodies show that this process is confined to clonemates or close relatives, whereas *Dictyostelium* spp. can form polyclonal fruiting bodies. Here, we examine kin recognition by co-culturing two distantly related *M. xanthus* strains under vegetative and starvation conditions. We show that strains segregate and antagonize via their type VI secretion systems (T6SS), yielding monoclonal fruiting bodies. In contrast, mixtures of T6SS mutant strains do not antagonize and form chimeric swarms and fruiting bodies with spores from both strains. Nevertheless, within these T6SS mutant swarms and fruiting bodies, strains segregate, thus revealing that nonlethal kin discrimination mechanisms also exist in this species. These findings further suggest that T6SS are a major mediator of lethal kin discrimination between distantly related strains. Thus, lethal kin discrimination ensures homogeneous populations and monoclonal fruiting body development, whereas nonlethal mechanisms play a more subtle and less discriminatory role similar to *Dictyostelium* spp.

## Graphical Abstract

**Figure:**
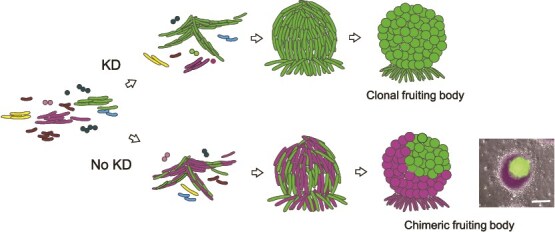

## Introduction

Multicellularity requires cells to cooperate to form functional tissues and for individuals to reach maturity. Failure to cooperate leads to diseases, such as cancer, or non-viability [1]. In animals and plants, multicellularity arises from a single-cell bottleneck, where fusion of gametes forms a zygote from which all subsequent cells are derived. This strategy ensures genetic uniformity across cells and enables purging of mutations that undermine cooperation. In contrast, aggregative multicellularity—where cells assemble from the environment to form multicellular structures—poses greater risks of genetic conflict due to the potential inclusion of nonclonal cells. Genomic conflicts among conspecific bacterial isolates are particularly high because of large differences in gene content, including social genes. For example, in bacterial species pangenomes, the number of accessory genes can exceed their core genome by orders of magnitude [2]. Therefore, unique genes or allele polymorphisms in social genes lead to conflicts that disrupt cooperation [3, 4]. These conflicts include exploitation by cheaters, where cells benefit from shared resources without contributing to their production [5–9].

For a developmental aggregation strategy to succeed, cells typically use mechanisms to distinguish self from nonself. This occurs by recognition and/or discrimination mechanisms. As defined here, kin recognition refers to cells that use genetic determinants to identify other cells that are clonal or highly related to conduct cooperative and beneficial acts. In contrast, we define kin discrimination as antagonistic acts directed at genetically dissimilar conspecifics. Two model systems that illustrate these concepts in aggregative multicellularity are the eukaryotic slime mold *Dictyostelium discoideum* and the social bacterium *Myxococcus xanthus*. Despite vast evolutionary divergence, both are soil-dwelling microbial predators that, upon starvation, aggregate into multicellular fruiting bodies composed of differentiated cell types, including stress-resistant spores.

In *D. discoideum*, kin recognition is primarily mediated by heterotypic interactions between polymorphic cell surface proteins TgrB1 and TgrC1 [10, 11]. Binding between compatible TgrB1/C1 allotypes triggers cytoskeletal rearrangements and motility-based strain segregation. Segregation occurs both early in aggregation and later during development, without direct antagonism or killing. TgrB1/C1 allorecognition also protects cooperative populations from cheater cells [12]. Cheating occurs during fruiting body development when one strain preferentially becomes spores as opposed to terminally differentiated stalk cells [8, 9, 13].

*Myxococcus xanthus* also uses kin recognition mediated by a polymorphic cell surface receptor called TraA and its cohort protein TraB. Self-recognition occurs by homotypic binding where specificity is determined by polymorphisms in TraA; hence recognition only occurs when cells have identical or nearly identical receptors [14–16]. When TraA–TraA recognition occurs, it leads to the bidirectional exchange of hundreds of different outer membrane proteins and lipids, in a process called outer membrane exchange (OME), which can confer benefits to kin [17–20]. However, we hypothesize that kin discrimination primarily drives the formation of homogeneous developmental populations from diverse environments, as previous studies have shown that conspecific strains intensely antagonize each other, likely obstructing multicellular development [21]. Kin discrimination mechanisms in myxobacteria include OME, which among its cargo are dozens of different polymorphic toxins that are delivered to neighboring cells, including non-clonal cells that happen to express a compatible *traA* allele [22, 23]. If the cells are clonal, they are protected because they express a cognate suite of immunity proteins that are not transferred. In contrast, OME between non-clonal cells results in mutual antagonism. Thus, OME has two layers of specificity required for social cooperation, the first being TraA–TraA recognition and the second by the exchange of a suite of polymorphic toxins. A second and broader kin discrimination system involves polymorphic toxin delivery by the type VI secretion system (T6SS), an injection platform evolutionarily related to phage tails [24]. Generally, the T6SS injects toxins into neighboring cells, and if cells are clonal, they are protected because they express a cognate set of immunity proteins that are not transferred. Studies by our lab and others showed that *M. xanthus* uses T6SS as a major kin discrimination mechanism against fellow myxobacteria [21, 25–27]. Similarly, other bacterial species also use T6SS in kin discrimination [4, 28, 29].

During starvation, over 10^5^  *M. xanthus* cells aggregate to build a fruiting body, yet only 1%–10% of cells differentiate into spores [30]. Other cells either persist as peripheral rods (~10%) or undergo massive lysis (~80%), making the system susceptible to exploitation by cheater cells that preferentially become spores. Laboratory studies show that developmentally deficient lines, evolved in asocial conditions, can outcompete ancestral strains for spore production when mixed [5]. Cheating has severe consequences, including drastically altering population dynamics or causing complete collapse of the social system and population extinction [31]. In nature, however, *M. xanthus* fruiting bodies are composed of nearly clonal cells [32], indicating that *M. xanthus* has mechanisms to determine genetic relatedness and exclude nonkin cells, which could consist of cheater cells.

Previously, we investigated kin discrimination between pairs of *M. xanthus* natural isolates grown under vegetative conditions [21]. These strains originated from a collection of 78 strains that were isolates from a 16 cm × 16 cm grid of forest soil [33]. Here, pairs of isolates were grown and spotted next to one another on agar surfaces and grouped into compatibility types based on the ability of swarming colonies to merge or not. In some cases, even isolates that were nearly genetically identical were incompatible [34]. Comparative genomics revealed that incompatible pairs harbored distinct prophage-associated genomic islands carrying unique polymorphic OME and T6SS toxin loci. Genetic inactivation of both systems abolished antagonism in many cases. However, some double mutants remained antagonistic. These strains each contained a large and unique Rhs toxin gene in prophage islands, and disruption of these loci, in combination with OME and T6SS mutations, eliminated residual killing [21].

In this study, we asked whether lethal kin discrimination systems are the only mechanisms that prevent cooperative behaviors, such as multicellular swarming and fruiting body formation, between genetically divergent *M. xanthus* strains, or whether nonlethal mechanisms also regulate cooperative behaviors. Specifically, we tested interactions between the environmental isolate A06 (from a German forest) and laboratory strain DK1622 (ancestor isolated in Ames, Iowa). These strains harbor incompatible *traA* alleles and thus cannot engage in OME. Consistent with this, when both strains were rendered T6SS-deficient, they no longer antagonized during vegetative growth and in response to starvation formed chimeric fruiting bodies with viable spores. The social interactions between the T6SS mutants are compared to those between the parental (wild-type) strains, revealing that nonlethal kin discrimination also exists. We further discuss the role of kin discrimination in the evolution of cooperation in aggregative multicellularity.

## Materials and methods

### Strain construction

Tables S1 to S3 list strains, plasmids, and primers used in this study. To create a labeled strain of DK1622, plasmid pMW106 (tdTomato cloned into pSWU30, oxytetracycline resistance) was electroporated and site-specifically recombined into the genome of DK1622 (wild type, WT) at the Mx8 *attB* site. To create labeled strains of A06, either plasmid pMW106 or pMW119 (sfGFP cloned into pKSAT, streptomycin resistance) was transformed and recombined at the Mx8 *attB* site of A06 or, similarly, to a previously constructed T6SS knockout (KO) mutant (gene disruption in the *tssA/vasJ* gene with a Km^R^ marker) of A06 [21]. A complementing *tssA/vasJ* strain was made by amplifying the WT gene and cloning the amplicon into pMR3487, which was then transformed and recombined at the plasmid integration site in DW2806 generating DW2815. Transformants were selected on CTT 1% agar media (1% casitone, 10 mM Tris–HCl [pH 8.0], 1 mM K_2_HPO_4_, 8 mM MgSO_4_, [pH 7.6]), supplemented with either 50 μg/ml kanamycin (Km), 10 μg/ml oxytetracycline (Tc) or 1.5 mg/ml streptomycin (Strep). Constructed strains were phenotypically characterized and, if necessary, validated by PCR or genomic sequencing.

### Growth and development

Strains were grown overnight to mid-exponential phase in CTT. For vegetative interactions, strains were resuspended to a density of 7.5 × 10^8^ cells/ml, mixed at a 1:1 ratio, and spotted on CTT 1% agar. At each time point, spots were collected, resuspended in liquid TPM, and cells were enumerated using fluorescence microscopy (see below). For starvation experiments on agar, cells were harvested and resuspended to a density of 3 × 10^9^ cells/ml and placed on TPM (10 mM Tris–HCl [pH 8.0], 1 mM K_2_HPO_4_, 8 mM MgSO_4_, [pH 7.6]) 1% agar plates. For submerged culture, strains were mixed 1:1 at a density of 3 × 10^7^ cells/ml and grown in 500 μl of CTT for 24 h in a 24-well plate. Afterward CTT was removed and 1 ml of MC7 starvation buffer (10 mM morpholinepropanesulfonic acid [pH 7.0] and 1 mM CaCl_2_) was added.

### Sequencing and phylogenetic analysis

For sequencing, strains A06 and DW2653, a derivative of A44 (this study), were grown overnight, and genomic DNA was harvested using the Wizard Genomic DNA Purification Kit (Promega). Nanopore sequencing and genome annotation were done by SeqCenter (Pittsburgh, PA), and the genome sequences were deposited to NCBI with accession numbers CP194063 (A06) and CP194062 (A44).

For phylogenetic analysis, eight complete *M. xanthus* genomes were obtained from Integrated Microbial Genomes (IMG) [35], as well as the complete A06 and A44 (DW2653) genomes. Using these 10 genomes, we performed multilocus sequence typing (MLST) analysis with seven housekeeping genes: *dnaA, gyrB, pyrG, rpoB, lepA, fusA,* and *secA.* Because 60 nucleotides were missing from the beginning of some *dnaA* sequences, these nucleotides were removed from the analysis. Sequences were aligned with Clustal Omega [36]. The aligned sequences were analyzed by ModelTest-NG [37] and the TIM2 + I + Gamma model of DNA substitution was selected. A maximum likelihood phylogeny was generated using RAxML-NG v1.1.0 [38] with this DNA substitution model and 10 000 transfer bootstrap expectation replicates.

To compare gene content between A06 and DK1622, genomes were uploaded to JGI IMG and analyzed using the Phylogenetic Profiler for Single Genes with the maximum E-value set to 1 × 10^−5^ and the minimum percent identity set to 50% [35]. ProgressiveMauve was used to compare genomes [39].

### Microscopy

For high magnification images of swarms, cells were spotted onto 1% agar CTT pads and imaged using an Olympus IX83 inverted microscope (40× objective) coupled to an ORCA-Flash 4.0 LT sCMOS camera and cellSens software. Low magnification images of swarms and fruiting bodies were captured using an Olympus SZX10 stereomicroscope coupled to a digital imaging system. Fluorescent images of fruiting bodies were captured with a Nikon E800 microscope (2× or 10× objectives) coupled to an ORCA-Flash 4.0 LT sCMOS camera and cellSens software.

### Spore assay

Strains were grown overnight in CTT at 33°C to early exponential phase, then resuspended to 3 × 10^9^ cells/ml in liquid TPM. Monocultures (10 μl) or 1:1 mixtures (20 μl) were spotted on TPM 1% agar plates. After 5 days of development, six spots were scraped from the plate and resuspended in TPM, heat treated at 50°C for 2 h to kill vegetative cells and sonicated to disperse spore clumps. Sonication was done on ice 3× for 30 s each with 20 s intervals. Following sonication, spores were serially diluted and plated on CTT with 2.5 μg/ml oxytetracycline or 500 μg/ml streptomycin.

## Results

### Genome comparisons between DK1622 and A06

**Figure 1:**
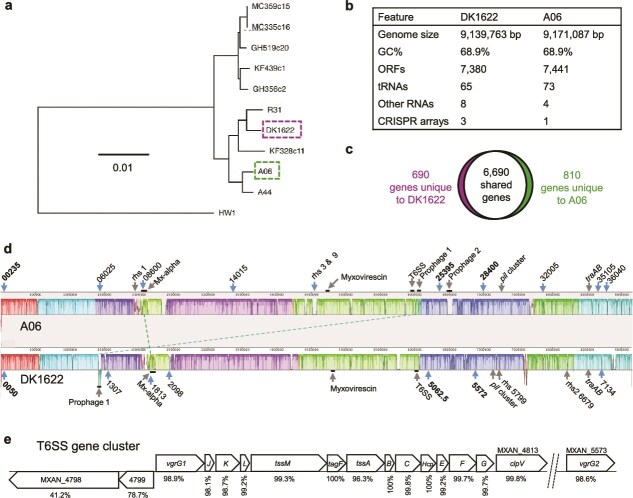
Phylogenic relationship between A06 and DK1622. (a) Maximum likelihood phylogeny showing the relationship of *M. xanthus* strains using seven conserved housekeeping genes: *dnaA, gyrB, pyrG, rpoB, lep, fusA*, and *secA.* Outgroup is *Myxococcus macrosporus* HW1. Bar represents number of substitutions per nucleotide site. (b) Genome comparison between strains. (c) Venn diagram comparing gene content between strains. (d) Alignment of A06 and DK1622 genomes using progressiveMauve. Areas of the same color represent homologous regions. Select loci shown. Numbers represent locus tags. Blue arrows indicate T6SS toxin-immunity loci where genes shared by both genomes (>97% ORF identity) are bold, whereas the other 10 toxin-immunity loci are unique to one genome. (e) T6SS structural gene cluster in DK1622, including accessory genes MXAN_4798 and MXAN_4799. A06 has an identical gene cluster and percent identities between genome ORFs are shown. ORFs with single letter designation follow the *tss* nomenclature.

In this study, we investigated interactions between two *M. xanthus* strains that were isolated on different continents, decades apart: environmental isolate A06 and the well-studied WT lab strain DK1622. To determine the evolutionary relationship between these and other *M. xanthus* strains, we conducted MLST of seven conserved housekeeping genes (Fig. 1a). This analysis was based on sequence identity across a concatenated 16,286 bp fragment. DK1622 grouped in a sister clade to A06, whereas A06 grouped with A44, an isolate from the same soil patch [33, 40], which shared 99.43% DNA sequence identity. In contrast, DK1622 shared 98.63% DNA sequence identity with A06. As expected, A06 and DK1622 16S rRNA genes were nearly identical, 99.93% with a single base difference, and had similar size genomes (Fig. 1b).

We expanded our analysis to include other fully assembled *M. xanthus* genomes (Fig. S1). We aligned these genomes with Mauve and found that overall, their genomes had very similar organization or synteny, including the large genomic islands for prophage Mx-alpha [41] and the myxovirescin polyketide biosynthetic gene cluster (Fig. S2), which are found in some but not all *M. xanthus* genomes (Fig. S1). In contrast, certain genomic islands were either absent in one strain or located in different chromosomal regions. A06 and DK1622 shared a large core genome, whereas 1500 or >10% of their genes were unique to each strain (Fig. 1c).

We next compared genes encoding cell surface-associated proteins (Fig. S3), as they are frequently social genes involved in cell–cell interactions and subject to polymorphisms. Genes encoding transport systems or biosynthetic enzymes, such as those involved in the T6SS, exopolysaccharide production (*eps*), and the developmental signaling protein *csgA* showed high conservation, sharing 98%–100% amino acid identity (Fig. S3). In contrast, proteins involved in surface recognition, including TraA*,* PilA*,* and PilY1.1*,* were divergent, with 78.86% to 82.25% amino acid identity, suggesting these strains belong to different social groups*.* Nearly all the sequence differences in TraA were concentrated in its variable domain, which governs the specificity of kin recognition [15, 16, 42]. As a result, the DK1622 and A06 TraA receptors were not compatible for binding and therefore cannot exchange polymorphic SitA toxins by OME [15, 21, 23].

Our prior work revealed that polymorphic effectors involved in kin discrimination were shuffled in diverse combinations in strains driven by horizontal gene transfer [21, 43]. To explore this further, we compared toxin-immunity proteins in A06 and DK1622 (Fig. S4). As expected, each genome contained a distinct repertoire of *sitA* (Table S4) and T6SS toxin loci. Of the 34 *sitA* genes found, only three sets showed significant identity (97% to 100%) between strains, suggesting their cognate immunity genes provide cross-resistance in those cases. An additional set of four had 80%–89% sequence identity, whereas other SitA proteins were more divergent. For T6SS effectors, of the 16 identified, only three sets showed high identity (98.9% to 99.7%), suggesting cross-resistance by their cognate immunity factors (Figs 1d and S4). Of the remaining 10 T6SS effectors, one pair was 89% identical and another was 74% identical between strains, thus reducing the likelihood of cross-resistance [44, 45]. The remainder were highly divergent (<65%, whereas most were <30% identical, Fig. S4). In contrast, the T6SS structural ORFs were highly conserved (98%–100%, Fig. 1e). BLAST searches of the T6SS toxins unique to A06 revealed close homologs in other myxobacteria (Table S5), indicating a role in kin discrimination. Additionally, some of these toxin-immunity cassettes were found on large prophage elements (Fig. S2), as described in other strains [21]. Finally, A06 and DK1622 each encoded unique *rhs* toxin loci that may contribute to kin discrimination (Fig. 1d). Given these divergent T6SS toxin repertoires, we predicted these strains would antagonize one another via T6SS.

### Vegetative antagonism mediated by T6SS

**Figure 2:**
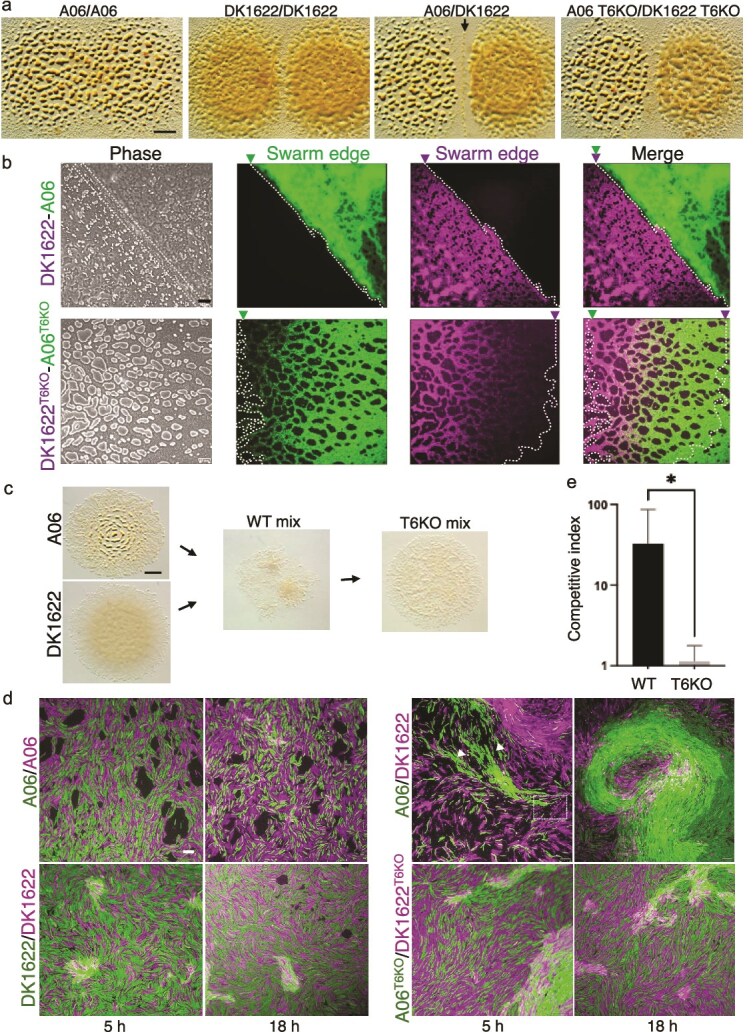
Social interactions between A06 and DK1622 derived strains. (a) Colony-merger incompatibility test. Aliquots of each strain were spotted next to one another on CTT 1% agar. Strains are WT or T6KO. Micrographs at 48 h. Arrow, demarcation line (incompatibility). Scale bar, 1 mm. (b) High magnification colony-merger assay on CTT 1% agar pad with indicated fluorescently labeled strains. Identical fields shown with different lighting sources. Dashed white lines outlines colony edges for each strain. Scale bar, 50 μm. (c) Aliquots of monocultures or 1:1 mixtures of indicated strains on CTT agar. Micrographs at 24 h. Scale bar, 1 mm. (d) Fluorescent micrographs of 1:1 strain mixtures at indicated times after spotting on 1% CTT agar pads. A06 and DK1622 derived strains labeled with GFP or tdTomato, respectively. Arrows and dashed box highlight spherical green and magenta cells poisoned in mixture. Scale bar, 10 μm. (e) Quantification of competition experiments of 1:1 mixtures of WT or T6KO strains. Mixtures spotted on CTT plates, collected at 24 h, and the number of cells from each strain was enumerated by fluorescence microscopy to determine their competitive index (ratio between strains compared to time 0). Asterisk indicates statistical significance based on unpaired *t-*test, ^*^P < 0.05. Error bars represent standard deviations from three biological replicas.

Inter-strain antagonism was tested by placing A06 and DK1622 cultures adjacent to one another on nutrient agar. As previously observed for non-clonal strains [34], a demarcation line formed between the swarms, whereas clonal swarms merged seamlessly (Fig. 2a). To determine whether antagonism was mediated by T6SS, we constructed T6SS deletion or gene disruption mutants (both referred to as T6KO) in both strains and repeated the assay. In this pairing, no demarcation was apparent. However, to confirm this latter finding, assays were repeated with differentially fluorescently labeled strains placed on glass slides coated with an agar pad (Fig. 2b). At these higher magnifications, the WT strains again formed a clear demarcation, whereas the T6SS mutants penetrated each other’s swarm fronts with no signs of cell lysis. These results suggest that T6SS activity underlies antagonism.

In a second assay, we mixed the WT strains together at a 1:1 ratio and transferred them on nutrient agar, alongside monoculture controls. After 24 h, the mixed cultures showed severe growth inhibition, in stark contrast to robust monoculture growth (Fig. 2c). In contrast, co-cultures of the corresponding T6SS mutants grew robustly, approaching monoculture levels, further indicating that antagonism was reduced or eliminated.

Strain antagonism was quantified using differentially labeled DK1622 and A06 strains with fluorescent markers. DK1622 expressed tdTomato and was resistant to oxytetracycline, whereas A06 expressed GFP and was resistant to kanamycin. Strains were mixed 1:1 and monitored by time-lapse fluorescence microscopy. By 2 h after mixing, morphological changes began to be seen and by 4 h 13.7% of the WT A06/DK1622 mixture cells had visibly lysed (Fig. S5). By 18 or 24 h, a major fraction of DK1622 cells had vanished or had morphologically compacted to spheres (Fig. 2d and e), indicating A06 was the dominant strain. As controls, isogenic strain mixtures of A06 or DK1622, expressing different fluorescent proteins, showed no signs of antagonism (Figs 2d and S5). To confirm that the T6SS from A06 was the cause of the dominant killing phenotype, the A06^T6KO^ strain was complemented with the corresponding ectopically expressed WT gene (*tssA/vasJ*), which indeed restored antagonism to DK1622^T6KO^ (Fig. S6).

The role of T6SS was assessed by conducting similar mixing experiments with differentially labeled T6KO mutants. At both 5 and 18 h, we observed no evidence of cell lysis, although there was a tendency for strains to segregate into groups of themselves, as compared to isogenic strain mixtures (Fig. 2d). The T6KO strain ratios remained relatively stable at 24 h (Fig. 2e). We conclude that under vegetative growth, antagonism between A06 and DK1622 was primarily, if not entirely, mediated by their T6SS, where A06 emerged as the dominant competitor.

### Chimeric fruiting bodies form in the absence of T6SS-mediated discrimination

**Figure 3:**
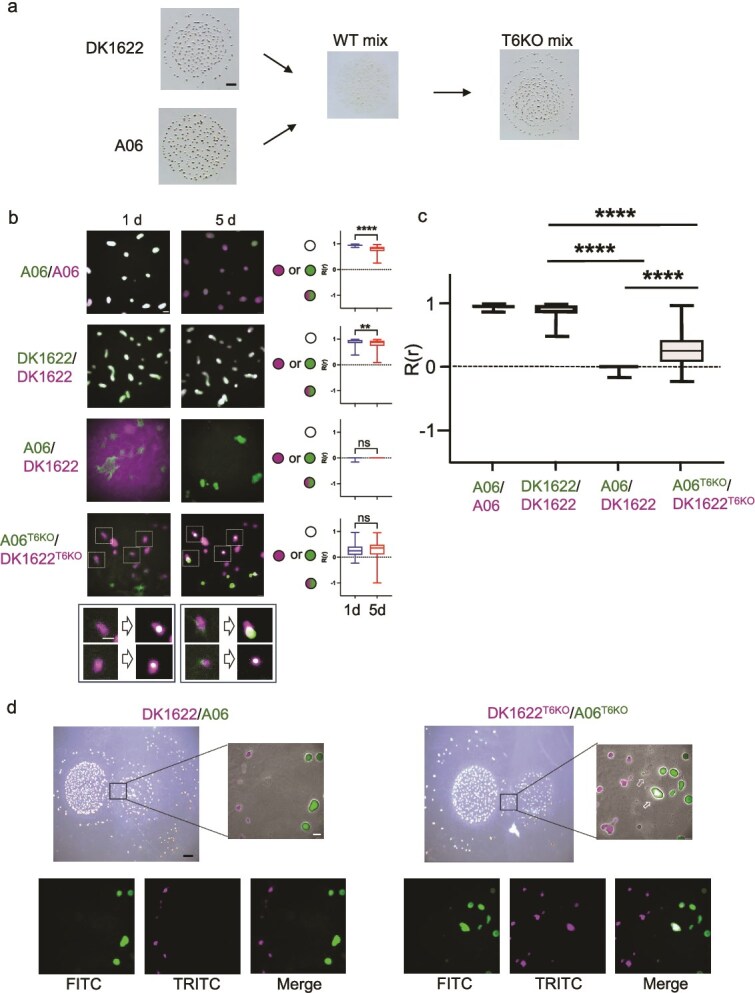
Developmental interactions between A06 and DK1622 derived strains. (a) Aliquots of monocultures or 1:1 strain mixtures on TPM 1% agar plates at 72 h. Dark spots represent mature fruiting bodies. Scale bar, 1 mm. (b) Fluorescent micrographs of 1:1 strain mixtures of strains on TPM 1% agar at two developmental days. Bottom enlarged fruiting body images of boxed areas at two different days. Scale bars, 50 μm. Right panels, Pearson’s correlation coefficients of fluorescently labeled fruiting bodies formed from 1:1 strain mixtures at 1 and 5 days on TPM 1% agar. Value of 1 indicates perfect correlation between fluorescent channels; a value of −1 indicates perfect segregation between fluorescent channels within a fruiting body. Asterisks indicate statistical significance based on Student’s *t-*test; ^**^P < 0.01, ^****^P < .0001, ns = not significant. Error bars represent standard deviations from three biological replicas. Bottom panels show enlarged images of boxed fruiting bodies from mixture of T6KO strains at 24 h and 5 days. Scale bar, 50 μm. (c) Comparison of Pearson’s correlation coefficients between strain mixtures from panel B at 24 h. Asterisks indicate statistical significance based on Student’s *t-*test; ^****^P < 0.0001. Error bars represent standard deviations. (d) Monocultures of WT or T6KO strains spotted next to one another on TPM agar after 1 week. Micrographs show colony interface. Arrows indicate chimeric fruiting bodies. Scale bars, 1000 μm and 500 μm.

To investigate developmental interactions, we placed a 1:1 mixture of WT strains on starvation agar and monitored development compared to monoculture controls (Fig. 3a). In WT mixtures, few mature fruiting bodies emerged after 5 days, which contrasted with monoculture development. In the T6KO mixture, fruiting body development was restored (Figs 3a and S7).

We used fluorescence microscopy to monitor development under a different condition. Submerged culture was used because it was amenable to extended duration time-lapse microscopy. Although WT A06 and DK1622 cells were initially well mixed, they spatially segregated within the first day (Fig. S8A). As time progressed, the A06 population increased, whereas the DK1622 population correspondingly decreased (Fig. S8B). Mature fruiting bodies appeared by Day 3, but they were few in number from WT mixtures compared to their monoculture controls. For the fruiting bodies that formed in the WT mixture, they fluoresced in only one channel, indicating they were derived from a single strain. In contrast, when the T6KO mutants were mixed in submerged culture, they formed more numerous and well-defined fruiting bodies that were mixtures of the two strains (Fig. S8).

Strains were also monitored under more favorable conditions for swarming and development, where fluorescently labeled T6KO mutants were mixed 1:1 and placed on starvation agar. Consistent with the above results, at 24 h fruiting bodies were more numerous than WT mixtures (Fig. 3b). Additionally, some fruiting bodies were chimeric, and as development progressed, strains segregated within fruiting bodies. Together, these results show that in the absence of T6SS-mediated killing, A06 and DK1622 co-develop to form chimeric fruiting bodies, although distinct spatial organization therein may still reflect individual strain identity.

We quantified the level of mixing within individual fruiting bodies by measuring fluorescent colocalization using Pearson’s correlation coefficient (Fig. 3b and c). In WT mixtures, most fruiting bodies at 24 h yielded a Pearson’s coefficient of 0, indicating fluorescence from only one channel and thus clonal composition. In contrast, fruiting bodies formed by T6SS mutant mixtures showed variable colocalization, with Pearson’s coefficients closer to +1, indicating chimerism. As development progressed, Pearson’s correlation coefficient decreased, indicating strains segregated as fruiting bodies developed (Fig. 3b). As controls, self-mixtures of GFP and tdTomato labeled DK1622 or A06 were assessed. As expected, Pearson’s correlation coefficient values were close to +1 throughout the experiment, indicating isogenic strains readily mixed and co-developed.

Cell dynamics during development were monitored by time-lapse fluorescence microscopy in submerged culture. A06 and DK1622 WT mixtures showed early spatial segregation and, over time, A06 spread and gradually overtook DK1622, resulting in aggregates composed almost exclusively of A06 (Movie S1). In contrast, T6KO mixtures of A06 and DK1622 showed extended periods (> 48 h) of co-migration, rippling, and aggregation (Movie S2). By the end of the experiment (5 days), the mutants formed chimeric fruiting bodies, like those mentioned above (Figs 3 and S8). In controls, self-mixtures of A06 or DK1622 showed thorough mixing, migration, and dynamic fruiting body development (Movies S3 and S4). Rippling was also evident, and for A06 it lasted for days.

To simulate more natural interactions, as opposed to forced mixing, we spotted labeled WT or T6KO cultures adjacent to one another on starvation agar and monitored them at the colony interface (Fig. 3d). WT swarms failed to merge, thus exhibiting nonself exclusion. In contrast, T6SS mutant swarms mixed at the interface, and chimeric fruiting bodies formed. Together, these results show that under starvation conditions, the T6SS mediates mutual antagonism where A06 ultimately dominates DK1622. In the absence of T6SS antagonism, strains co-develop and form chimeric fruiting bodies.

### Strain segregation during vegetative motility and growth

**Figure 4:**
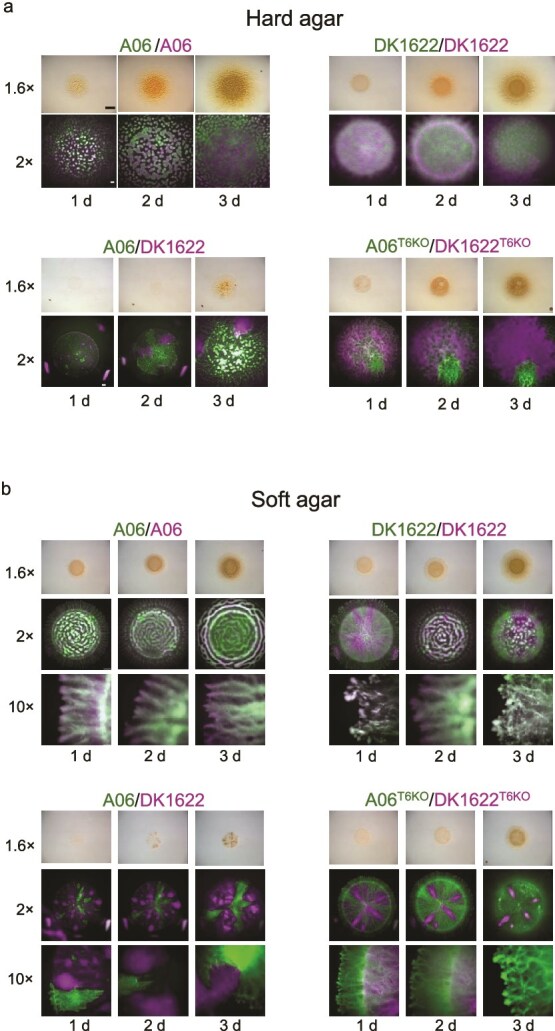
Swarming behavior of strain mixtures on hard and soft agar. (a) 1:1 strain mixtures spotted on CTT 1% agar. Micrographs of the same spot at different magnifications and at different times. Scale bars for 1.6× = 5 mm, for 2× = 500 μm, and for 10× = 100 μm. (b) Same as (a) except mixtures spotted on 0.5% CTT agar.

Strain behaviors were observed during vegetative growth where mixtures were spotted on rich media with either hard or soft agar composition, to simulate two environmental conditions, which promote adventurous (A) motility, driven by focal adhesion complexes, or social (S) motility, driven by retracting type IV pili, respectively [46, 47]. On both hard and soft agar, isogenic control mixtures of DK1622 or A06 were generally well mixed (Fig. 4), where A06 swarmed at a faster rate than DK1622 (Fig. S9). In the WT A06 and DK1622 mixtures, on hard and soft agar, minimal growth was visible after 1 and 2 days, reflecting mutual antagonism (Fig. 4a and b). By Day 3 on hard agar, A06 had expanded and mostly annihilated DK1622, except for sectored flares at the colony edge. By Day 3 on soft agar, WT DK1622 fared better than on hard agar mixtures, but again the strains segregated into distinct patches (Fig. 4b). In the T6KO mixtures, on both hard and soft agar, growth was robust, where strain segregation was seen on Day 1, and their segregation pattern intensified and persisted throughout the experiment. Additionally, in mixtures of both WT and T6KO strains, swarm areas were reduced (compared to monoculture controls), indicating these strains inhibited each other’s motility. In summary, although the T6KO mutations allowed A06 and DK1622 to co-exist during vegetative swarming, the strains nevertheless partitioned into distinct sectors.

### T6SS mutants restore sporulation efficiency between A06 and DK1622 mixtures

**Figure 5:**
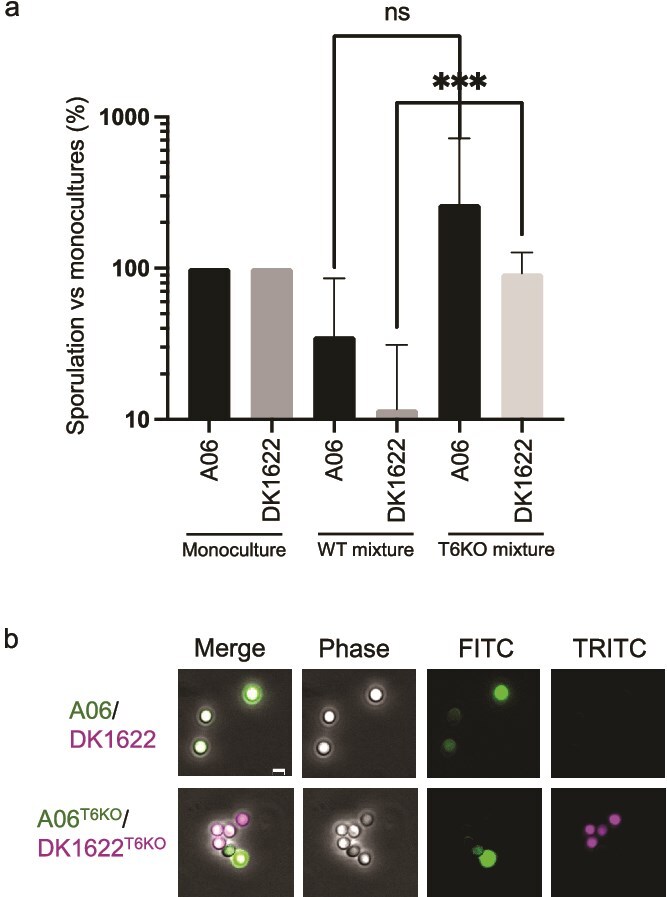
Sporulation compatibilities of strain mixtures. (a) Sporulation efficiency of each strain in 1:1 mixtures relative to monoculture development after 5 days. Asterisks indicate statistical significance based on two-way ANOVA; ^***^P = 0.0003, ns = not significant. Error bars represent standard deviations from three biological replicas. (b) Fluorescent micrographs of spores harvested from fruiting bodies after 1 week of development on TPM agar. Scale bar, 2 μm.

We investigated sporulation efficiencies of WT and T6KO mixtures on starvation agar, relative to controls. In the WT mixtures, the sporulation efficiencies were lower than their respective monocultures, apparently caused by mutual antagonism (Fig. 5a). In the T6KO mixtures, sporulation levels returned to around monoculture levels, indicating that these divergent strains cooperated during development. Additionally, spherical retractile spores were seen from both strains in the T6KO mixture (Fig. 5b).

## Discussion

### T6SS is the major kin discrimination determinant between A06 and DK1622

In a previous study, we found that OME, T6SS, and, in certain strains, Rhs systems all function in kin discrimination between conspecific strains [21]. Here, we report that T6SS is the dominant mechanism of kin recognition between two distantly related *M. xanthus* strains. In the case of OME-mediated antagonism, it requires homotypic binding by partnering cells with compatible TraA receptors. Because TraA is highly polymorphic [15], the likelihood that two isolates contain compatible *traA* alleles is relatively low. In contrast, T6SS provides a broad and effective mechanism for kin discrimination, because toxin delivery is unidirectional and *M. xanthus* lacks a known specificity mechanism to target cells. In other species, T6SS functions in intraspecific and interspecific competition, as found in *Vibrio* spp. [28, 48–50] and *Serratia marcescens* [51]. Aside from the T6SS and the unique toxin-immunity loci between A06 and DK1622, these strains also have different genomic islands, which include *rhs* genes, so we cannot rule out that other systems may play a minor discriminating role. Additionally, A06 and DK1622 each contain hundreds of unique accessory genes (<50% identity), so, in principle, they could also contribute toward social interactions.

We found that although there is mutual antagonism, A06 uses its T6SS to dominate DK1622 under both vegetative and developmental conditions. Several explanations may account for this result. These strains may differ in their T6SS firing dynamics, with A06 deploying its T6SS faster or more frequently, or A06 may produce more T6SS complexes per cell. Other possibilities include that A06 toxins are more potent or A06 has superior cross-immunity toward DK1622 T6SS toxins and/or A06 partially blocks toxin entry [44, 45]. Finally, because A06 swarms at a faster rate, A06 may better evade T6SS attacks and/or more quickly hunt down its targets.

### Chimeric development in T6KO mixtures

**Figure 6:**
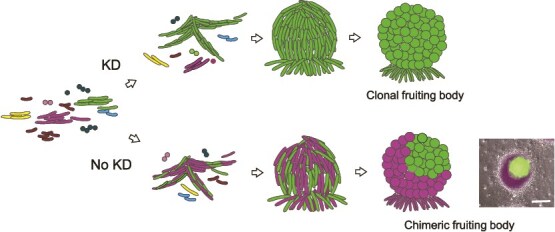
Model for the role of kin discrimination (KD) during fruiting body development. Rods represent vegetative myxobacteria cells; other shapes represent unrelated soil microbes. Cells of the same color represent kin or clonal cells. During KD (top), cells of one kin group dominate in aggregates to form a monoclonal fruiting body. Nonkin minority cells are eliminated from the collective. In the absence of KD (bottom), distinct strains aggregate and form a chimeric fruiting body where cells subsequently segregated. Right, micrograph of chimeric fruiting body between A06-T6KO (green) and DK1622-T6KO (magenta) from Fig. S8. Scale bar, 50 μm.

Under developmental conditions, the results of WT strains mirrored those during vegetative conditions—A06 dominated and nearly all fruiting bodies were composed entirely of A06. In the absence of T6SS antagonism, chimeric fruiting bodies formed that produced viable spores from both strains (Fig. 6). Early on these chimeric fruiting bodies were well-mixed, but over the course of several days, strains within fruiting bodies segregated to distinct regions. The mechanism that drives segregation is unknown but likely involves cell-surface molecules and motility, which likely also explains segregation seen during vegetative conditions. Candidates involved in segregation are the motility proteins PilA and/or PilY1.1, because they show significant sequence differences and type IV pili power S-motility and are implicated in kin recognition in other species [52, 53].

Strain mixing experiments were also done in the eukaryotic social amoeba *D. discoideum*. Here, isolates of varying degrees of relatedness were mixed to determine their ability to form chimeric fruiting bodies [54]. In that system, polymorphic adhesion molecules drove strains to segregate within fruiting bodies, analogous to our T6SS mutant findings. Further, they found that as the phylogenetic distance between strains increased, the greater their degree of segregation [54]. In natural and experimental settings, wild *Dictyostelium* spp. are able to form polyclonal fruiting bodies [55–57], which contrast with our lethal *M. xanthus* kin discrimination findings as well as natural isolate findings [32]. Thus, lethal kin discrimination in myxobacteria contrasts with its absence in social slime molds. In future *M. xanthus* studies, it would be of interest to test whether degrees of strain relatedness impact degrees of strain segregation when lethal discriminating mechanisms are absent.

In WT mixtures, the sporulation efficiency of both strains was reduced due to mutual antagonism. With T6KO mixtures, A06 sporulated more efficiently than as a monoculture. This finding aligns with other studies showing that some strains sporulate more efficiently in mixtures, likely due to exploitation [58]. In contrast, the sporulation efficiency of DK1622 was nearly identical in T6KO mixtures as in monoculture, indicating that any chimeric benefit was limited to A06. One candidate mechanism to explain this synergy could be C-signal, whose transmission is cell–cell contact dependent and is involved in developmental cheating [59, 60]. During development, threshold C-signal concentrations are needed to trigger aggregation and sporulation. How C-signal is perceived is unknown, but once sufficient C-signal is present, developmental gene expression proceeds to initiate sporulation [61]. The C-signal is a product of the *csgA* gene [61], which is 98.8% identical between the two strains, so presumably the same C-signal is functional in both strains. Thus, A06 may be more sensitive to the C-signal and may require a lower concentration to initiate sporulation. However, there are numerous other possibilities to explain A06’s modest sporulation increases when mixed as T6KO mutants.

### Strain segregation during swarming

Both WT strain mixtures and T6KO mixtures segregated on hard and soft agar within the first 24 h. Although the underlying mechanism of segregation is not known, a plausible explanation is that strains produce a surface molecule that preferentially binds to itself, where again PilA and/or PilY1.1 are candidates because their alleles are divergent. Studies in *Vibrio* have shown that cells expressing the same pilin allele aggregate together, leading to segregation from strains expressing a different allele [52, 53]. Self-aggregation is also proposed as a mechanism that allows cells to defend themselves from rival T6SS attacks. Strain segregation may also be maintained by the “corpse barrier effect,” in which dead cells at the interface between strains prevent further mixing [53, 62].

Mixtures of either WT or T6SS mutants had reduced swarm rates on hard and soft agar compared to their monocultures. For the WT mixture, mutual antagonisms are a likely explanation. Although the T6KO strain mixtures showed increased swarm rates relative to WT mixtures, they lagged monoculture swarms. This reduction might be caused by residual antagonism by another mechanism. Alternatively, the polymorphic nature of PilA/PilY1.1 may disrupt interactions between divergent type IV pili and hence hinder swarm expansion. Future studies tracking cell and swarm movements where the *pilA/pilY1.1* alleles are swapped between strains would help address this hypothesis and to test their possible role in nonlethal kin discrimination.

## Supplementary Material

Supplementary_materials_wrag136

## Data Availability

All data are present in the main text, supplementary materials, or deposited at NCBI.
