# Supplementary material for: Aggregative chimeric multicellularity in the absence of lethal kin discrimination
Source: ISME J. 2026 Jun 11;20(1):wrag136. doi: 10.1093/ismejo/wrag136 (PMC13310138; doi:10.1093/ismejo/wrag136)
Supplement: Supplementary_materials_wrag136 [file supplementary_materials_wrag136.zip › Supplemental Material.docx]

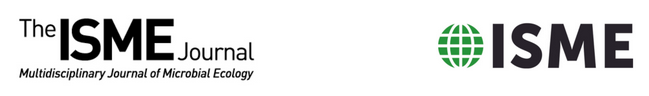


**Supplemental Material**

Aggregative chimeric multicellularity in the absence of lethal kin discrimination

Michael L. Weltzer, Pravas C. Roy, Jack Govaerts**,** and Daniel Wall

Department of Molecular Biology, University of Wyoming, 1000 E University Avenue, Laramie, WY, USA.


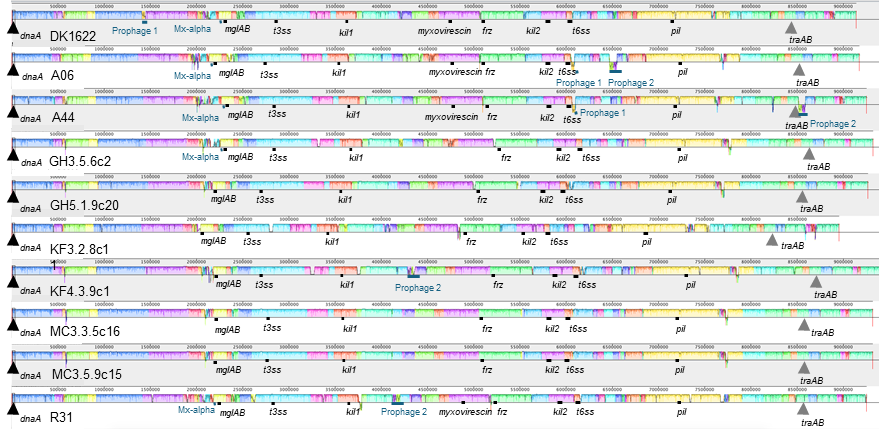


Figure S1 Mauve alignment of complete M. xanthus genomes. Colored blocks represent homologous regions shared between genomes. Select elements and loci shown.


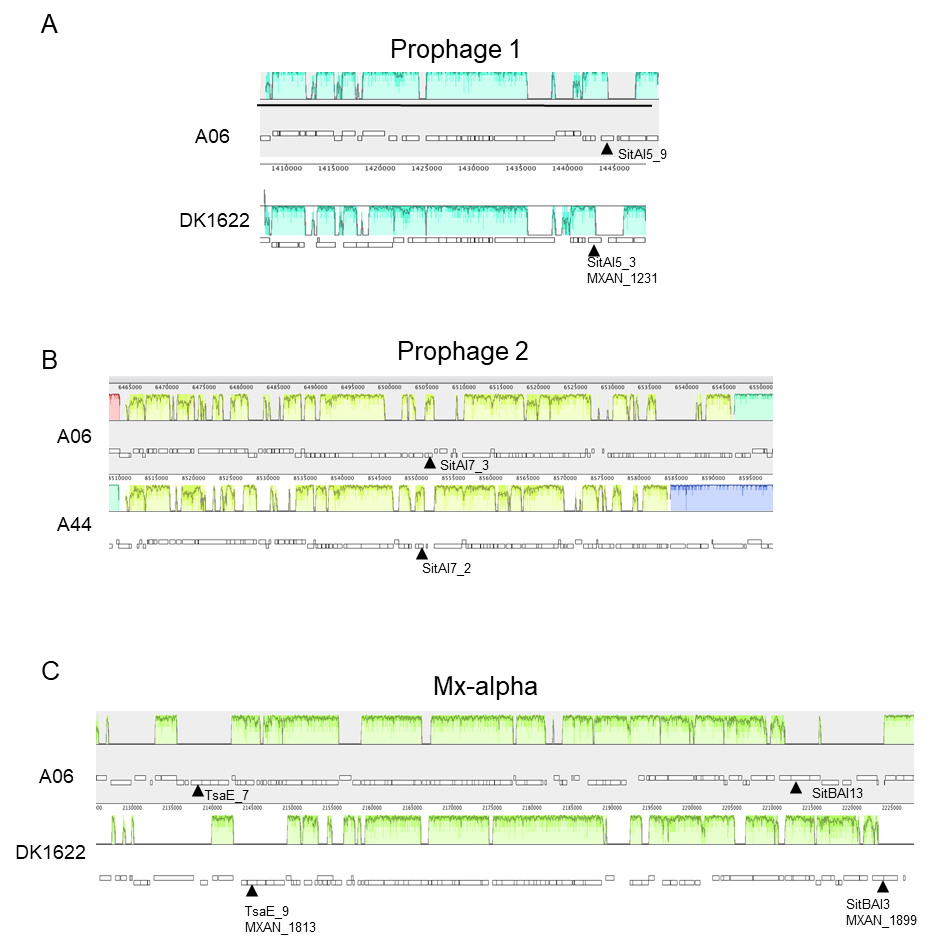


Figure S2 Mauve prophage alignment comparisons. (A) Prophage 1 in A06 and DK1622. (B) Prophage 2 (yellow) in A06 and A44 (DK1622 lacks this element). (C) Mx-alpha elements in A06 and DK1622. Triangles indicate T6SS and *sitA* toxin loci. Additional details in [1].

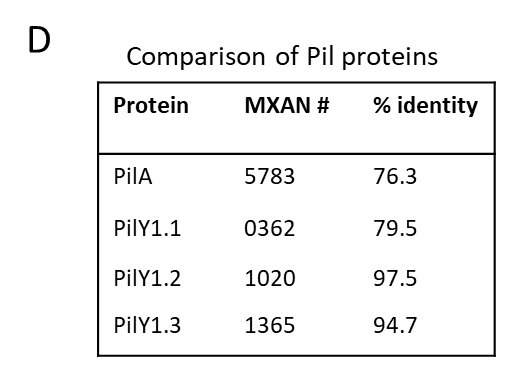
 PilY1.1


 PilA

Figure S3 Analysis of cell surface proteins. (A) Alignment of DK1622 and A06 TraA protein sequences. (B) Cartoon depicts homotypic binding between compatible TraA receptors, leading to outer membrane exchange. Bottom, domain architecture of TraA and TraB with sequence identities between strains. (C) Mauve alignment of the exopolysaccharide (EPS) gene cluster (MXAN_7415–7451) between strains; bottom predicted ORF functions, adapted from [2]. (D) Table compares protein identities between A06 and DK1622 and protein alignments of PilA and PilY1.1 shown.


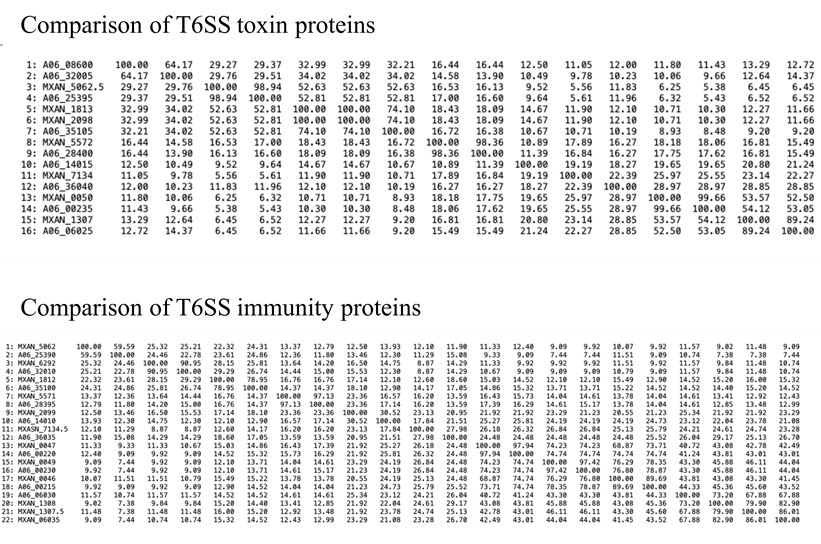


Figure S4 Comparison of T6SS effectors and immunity proteins. Matrixes show percent protein identity of T6SS effectors and immunity proteins between strains A06 and DK1622 (MXAN). The additional immunity genes (22) compared to toxin genes (16), may confer resistance to other toxins [3, 4]


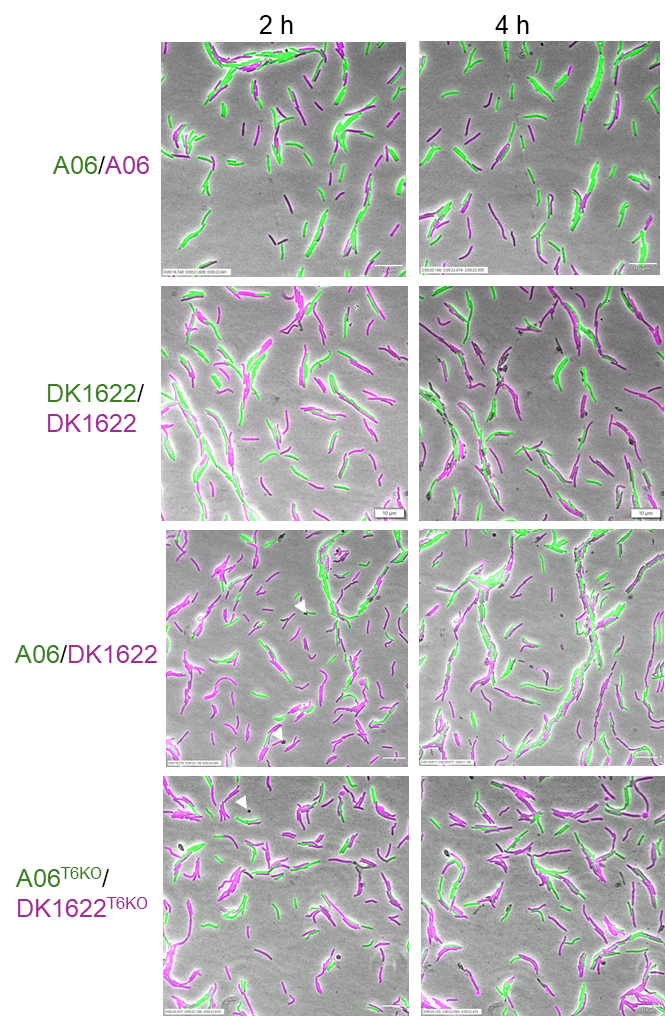


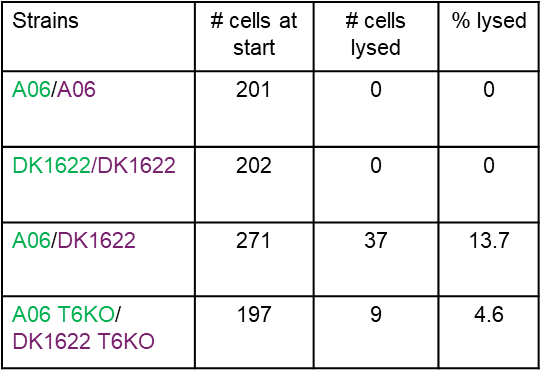


Figure S5 Strain antagonism alleviated by T6SS mutations. Micrographs from time-lapse movies of strain mixtures on 1% agar pads at indicated times after spotting. Morphological changes and cell debris are visible (triangle). Scale bar, 10 μm. Table summarizes the number and percentage of cells lysed by following time-lapse movies of individual cells for 4 h.


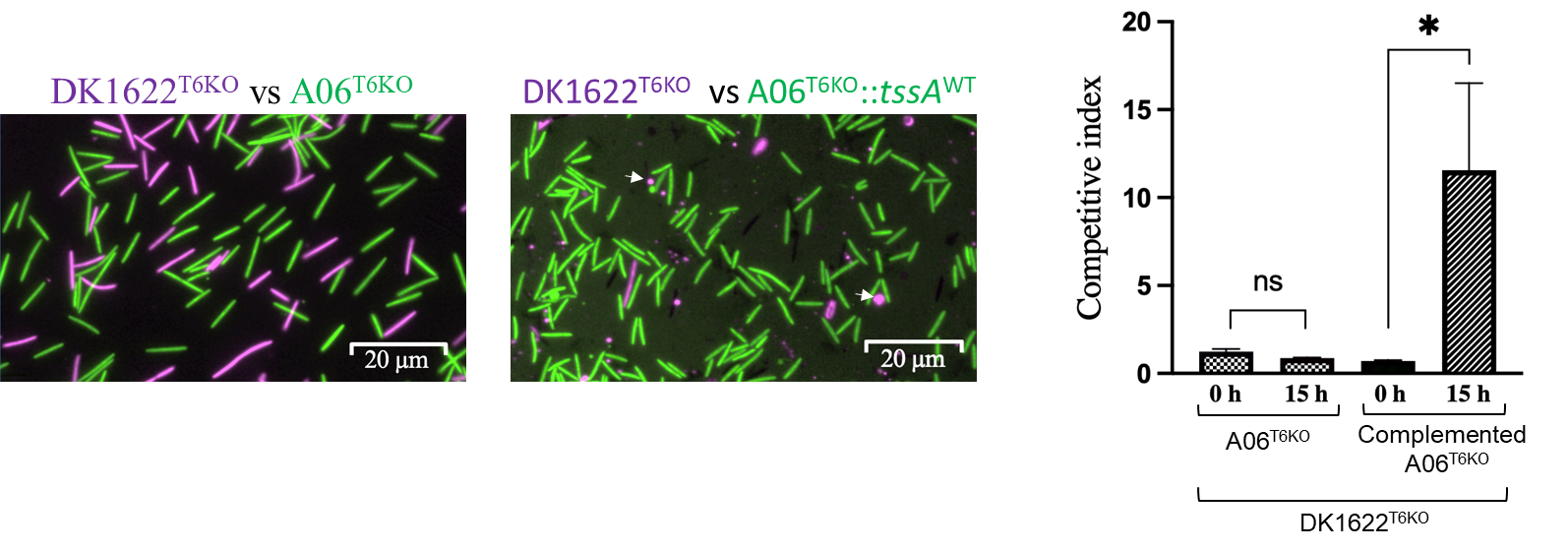


Figure S6 Complementation of the A06^T6KO^ restores DK1622^T6KO^ antagonism. A06^T6KO^ contains a gene disruption in *tssA*/*vasJ* with an integrated kanamycin marker. This strain was transformed with a chromosomal ectopically expressed *tssA*/*vasJ* WT gene. Left panels, the indicated strains were mixed 1:1 and placed on agar for 15 h and then collected and imaged (white arrows, rounded lysed DK1622^T6KO^ cells, tdTomato labeled). Right, cells counted at indicated times and the competitive index for A06 strains calculated. Asterisk indicates statistical significance based on a paired t-test; *P < 0.05, *n* = 3, >4,000 cells counted at each time point.


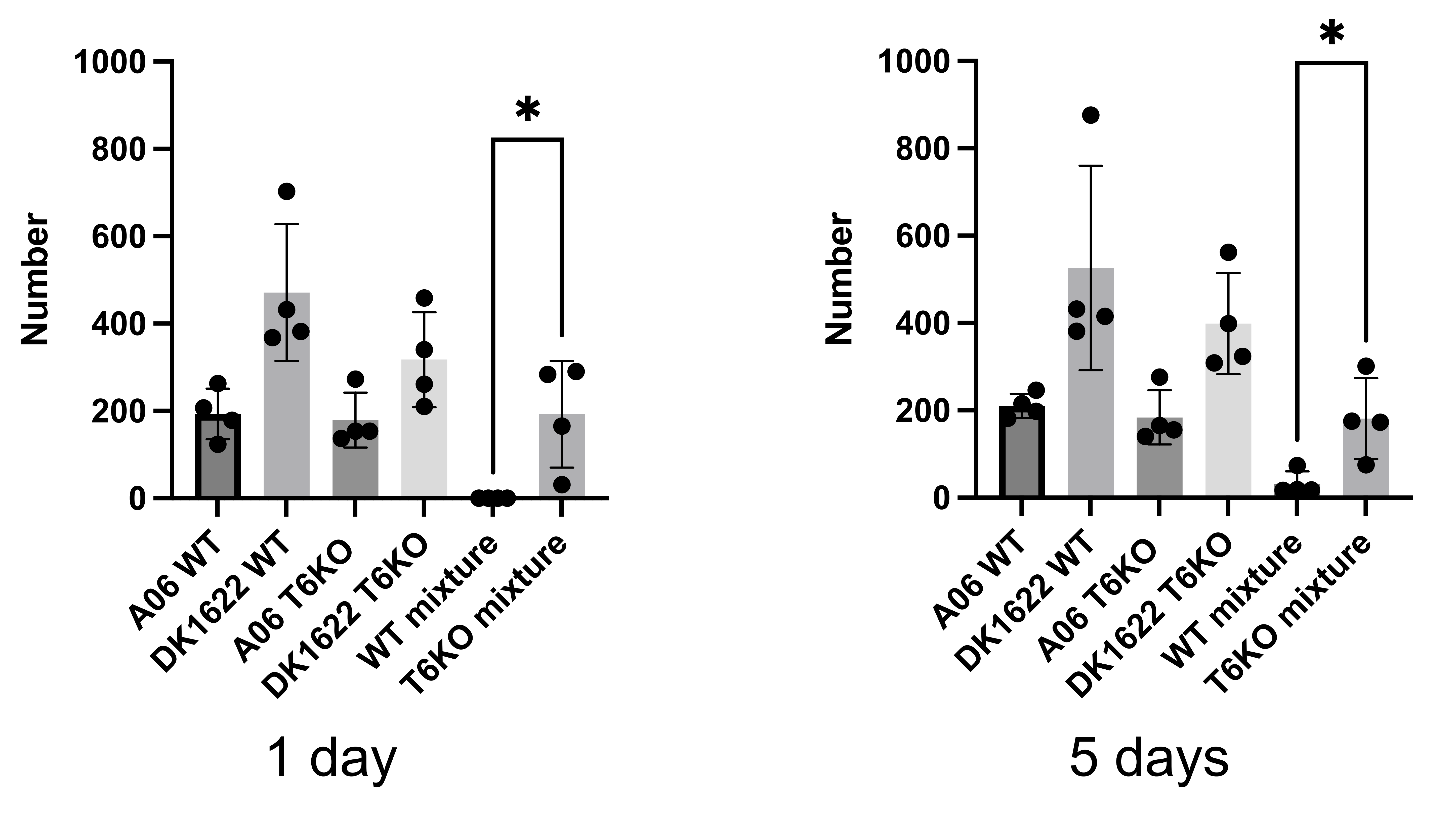


Figure S7 T6KO mutations restore fruiting body development. Fruiting body number quantified at 1 and 5 days in 20 μL spots of monocultures and strain mixtures on TPM agar. Asterisks indicate statistical significance based on one-way ANOVA; *P < 0.05, *n* = 3.


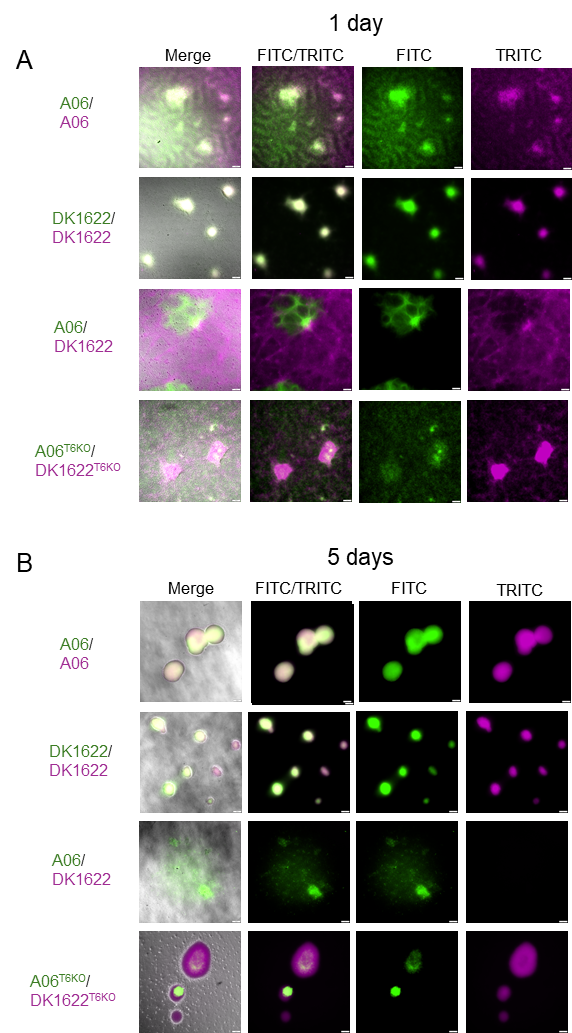


Figure S8 Chimeric fruiting bodies form between T6KO strain mixtures. Micrographs from submerged cultures after addition of starvation buffer at day 1 (A) and day 5 (B). Scale bars, 50 μm.


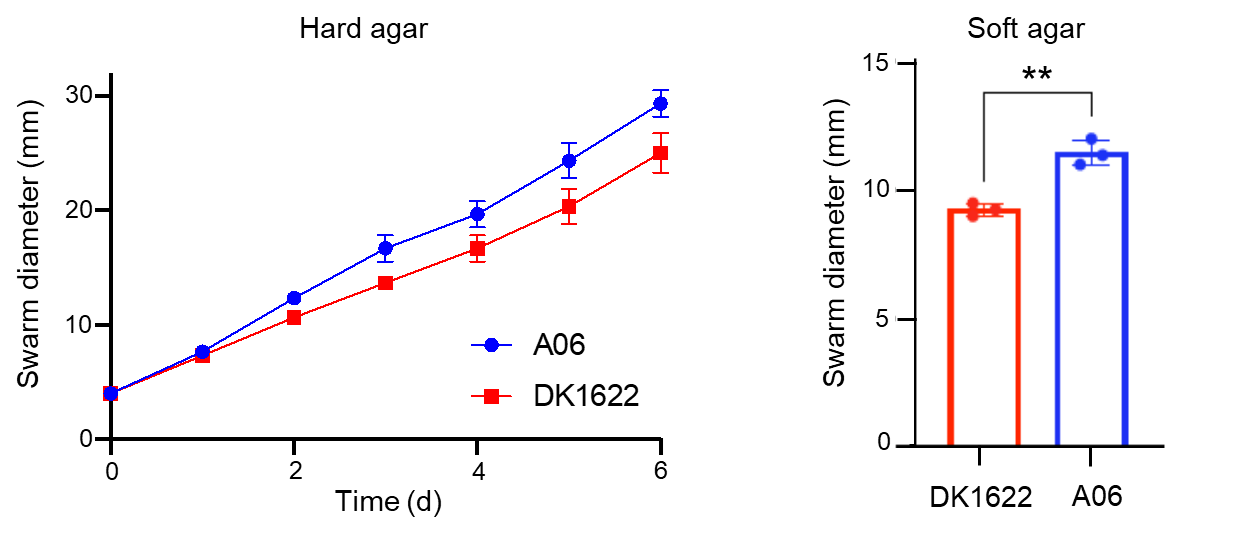


Figure S9 Swarm rates of A06 and DK1622. Swarm diameters of 5 μL monoculture spots measured over six days. Error bars represent standard deviations, *n* = 3. Right, unpaired t test with Welch's correction *P* value 0.007 after six days.

Table S1 Strains used in this study

| **Strain** | **Description** | **Reference** |
| --- | --- | --- |
| DK1622 | WT lab strain, *M. xanthus* | [5] |
| A06 | WT, *M. xanthus* | [6] |
| DW2445 | DK1622 P_3,4_-eGFP Km^R^ | [7] |
| DW2803 | DK1622 tdTomato (pMW106) | This study |
| DW2671 | DK1622 ΔT6SS (Δ4800-4813) P_IPTG_-tdTomato Tc^R^ | [8, 9] |
| DW2804 | A06 tdTomato (pMW106) | This study |
| DW2805 | A06 sfGFP (pMW119) | This study |
| DW2472 | A06 *tssA*::TOPO XL, Km^R^ (pAD162) | [1] |
| DW2806 | DW2472 sfGFP Strep^R^ (A06 T6KO GFP; pMW119) | This study |
| DW2815 | DW2806 pPR123 Tc^R^ (*tssA*^WT^) | This study |

Table S2 Plasmids used in this study

| **Plasmid** | **Relevant Properties** | **Source** |
| --- | --- | --- |
| pKSAT | *M. xanthus* expression vector, Mx8 *attP/int*, Strep^R^ | [10] |
| pSWU30 | *M. xanthus* expression vector, Mx8 *attP/int*, derived from pSWU19, Tc^R^ | [11] |
| pMR3487 | *M. xanthus* expression vector, IPTG-inducible promoter, 1.38 kb chromosomal integration site | [12] |
| pMW106 | pSWU30 P_IPTG_ (Δ*lacI*) tdTomato, Tc^R^ | This study |
| pMW119 | pKSAT P_R3/4_ sfGFP, Strep^R^ | This study |
| pAD162 | pCR TOPO XL A06 *tssA* (*vasJ*) internal fragment, Km^R^ Zeo^R^ | [1] |
| pPR123 | pMR3487-*tssA*^WT^ at XbaI/KpnI sites | This study |

Table S3 Primers used in this study

| **Primer** | **Sequence (5’ - 3’)** |
| --- | --- |
| sfGFP.fwd | AATTAGATGGTGATGTTAATGG |
| sfGFP.rev | AGTTACAAACTCAAGAAGG |
| tdTomato_fwd | AGCAAGGGCGAGGAGGTCATC |
| tdTomato_rev | CCTTGGAGCCGTACATGAACTGG |
| tssA.fwd | ATTAATTCTAGAGC**ATG**GGGTTCACTGACATGGCAGTGCAGTCGCC |
| tssA.rev | ATTAATGGTACCCGCGGTACGACGGGTGTCTTTCAAGCG |

Restriction sites underline, start codon bold

Table S4 SitA toxins comparisons

| A06 locus tag | DK1622 locus tag | % identity | SitA family |
| --- | --- | --- | --- |
| 9045 |  |  | 1/2 |
|  | 1899 |  | 3 |
|  | 4478 |  | 4 |
| 10570 |  |  | 4 |
| 560 | 119 | 97.1 | 5 |
|  | 485 |  | 5 |
|  | 648 |  | 5 |
| 24200 | 1231 | 88.2 | 5 |
|  | 1255 |  | 5 |
| 21625 | 4323 | 89.3 | 5 |
|  | 4844 |  | 5 |
| 33050 | 6511 | 81.9 | 5 |
|  | 7256 |  | 5 |
|  | 7453 |  | 5 |
| 20680 |  |  | 5 |
|  | 242 |  | 6 |
|  | 253 |  | 6 |
| 4995 | 1054 | 88.9 | 6 |
|  | 1544 |  | 6 |
| 12085 | 2496 | 97.5 | 6 |
| 29740 | 5843 | 100 | 6 |
|  | 6330 |  | 6 |
|  | 6448 |  | 6 |
|  | 7411 |  | 6 |
| 4995 |  |  | 6 |
| 32210 |  |  | 6 |
| 32736 |  |  | 6 |
| 2911 |  |  | 6 |
|  | 598.5 |  | 7 |
|  | 6560 |  | 7 |
| 26110 |  |  | 7 |
| 18740 |  |  | 7 |
| 18545 |  |  | 7 |
| 35336 |  |  | 7 |

Table S5 A06 T6SS effectors absent in DK1622 and present in other M. xanthus genomes

| **A06 locus tag** | **Name** | ***Myxococcus*  genomes** | **% identity** |
| --- | --- | --- | --- |
| 08600 | TsaE7 | GH3.5.6c2 | 99 |
| 14015 | AHH1 | sp. AB022 | 99 |
|  |  | sp. CA027 | 99 |
|  |  | sp. CA006 | 99 |
|  |  | sp. CA023 | 99 |
|  |  | sp. AB025A | 99 |
|  |  | sp. AB024B | 99 |
|  |  | sp. CA010 | 99 |
|  |  | sp. CA018 | 99 |
|  |  | sp. AB056 | 98 |
|  |  | sp. AB036A | 97 |
|  |  | KF3.2.8c11 | 97 |
|  |  | GH5.1.9c20 | 97 |
| 32005 | TsaE2 | KF3.2.8c11 | 96 |
| 35105 | TsaE11 | KF3.2.8c11 | 94 |
|  |  | sp. AB056 | 93 |
| 36040 | AHH6 | KF.3.9c1 | 96 |
|  |  | MC3.3.5c16 | 96 |
|  |  | GH3.5.6c2 | 96 |
|  |  | GH5.1.9c20 | 96 |
|  |  | MC3.5.9c15 | 96 |
|  |  | sp. NMCA1 | 96 |
|  |  | KF2.2.8c11 | 90 |

Supplemental movies

Movie S1 Time-lapse of A06-GFP/DK1622-tdTomato in submerged culture from 27 to 43 h after MC7 starvation buffer addition. Strain A06 overtakes DK1622. Frames taken every 30 s with 10× objective.

Movie S2 Time-lapse of A06 T6KO-GFP/DK1622 T6KO-tdTomato in submerged culture from 27 to 43 h after MC7 starvation buffer addition. Cell rippling occurs throughout the movie. Frames taken every 30 s with 10× objective.

Movie S3 Time-lapse of A06-GFP/A06-tdTomato in submerged culture from 27 to 43 h after MC7 starvation buffer addition. Cell rippling occurs throughout the movie. Frames taken every 30 s with 10× objective.

Movie S4 Time-lapse of DK1622-GFP/DK1622-tdTomato in submerged culture from 27 to 43 h after MC7 starvation buffer addition. Large fruiting bodies present. Frames taken every 30 s with 10× objective.

**References**

1. Vassallo CN, Troselj V, Weltzer ML, et al. Rapid diversification of wild social groups driven by toxin-immunity loci on mobile genetic elements. *ISME J,* 2020. 14(10): p. 2474-2487.

2. Perez-Burgos M, Garcia-Romero J, Jung J, et al. Characterization of the exopolysaccharide biosynthesis pathway in *Myxococcus xanthus.* *J Bacteriol*, 2020. **202**(19): p. e00335-20.

3. Kirchberger, PC, Unterweger D, Provenzano D, et al. Sequential displacement of type VI secretion system effector genes leads to evolution of diverse immunity gene arrays in *Vibrio cholerae.* *Sci Rep*, 2017. **7**: p. 45133.

4. Wang F, Luo J, Zhang A, et al. Differential crosstalk between toxin-immunity protein homologs divides *Myxococcus* nonself siblings into close and distant social relatives*.* *mBio*, 2025. **16**: p. e03902-24.

5. Wall D, Kolenbrander PE, Kaiser D. The *Myxococcus xanthus pilQ (sglA) g*ene encodes a secretin homolog required for type IV pilus biogenesis, social motility, and development*.* *J Bacteriol*, 1999. **181**(1): p. 24-33.

6. Vos M, Velicer GJ. Genetic population structure of the soil bacterium *Myxococcus xanthus* at the centimeter scale. *Appl Environ Microbiol*, 2006. **72**(5): p. 3615-25.

7. Vassallo CN, Cao P, Conklin A, et al. Infectious polymorphic toxins delivered by outer membrane exchange discriminate kin in myxobacteria. *eLife*, 2017. **6**: p. e29397.

8. Chang YW, Chen S, Tocheva EI, et al. Correlated cryogenic photoactivated localization microscopy and cryo-electron tomography*.* *Nat Methods*, 2014. **11**(7): p. 737-9.

9. Troselj V, Treuner-Lange A, Sogaard-Andersen L, et al. Physiological heterogeneity triggers sibling conflict mediated by the type VI secretion system in an aggregative multicellular bacterium*.* *mBio*, 2018. **9**(1): p. e01645-17.

10. Nariya H, Inouye, M. MazF, an mRNA interferase, mediates programmed cell death during multicellular *Myxococcus development.* *Cell*, 2008. **132**(1): p. 55-66.

11. Wu SS, Kaiser D. Genetic and functional evidence that type IV pili are required for social gliding motility in *Myxococcus xanthus.* *Mol Microbiol*, 1995. **18**(3): p. 547-58.

12. Iniesta AA, Garcia-Herase F, Abellon-Ruiz J, et al. Two systems for conditional gene expression in *Myxococcus xanthus* inducible by isopropyl-beta-D-thiogalactopyranoside or vanillate. *J Bacteriol*, 2012. **194**(21): p. 5875-85.
